# Effects of rural-to-urban migration on healthcare utilization of middle-aged and older adults: evidence from the China health and retirement longitudinal study

**DOI:** 10.3389/fpubh.2025.1576285

**Published:** 2025-04-14

**Authors:** Lu Xu, Yingchun Chen

**Affiliations:** ^1^Department of Health Management, School of Medicine and Health Management, Tongji Medical College, Huazhong University of Science and Technology, Wuhan, China; ^2^Key Research Institute of Humanities and Social Sciences of Hubei Provincial, Department of Education, Research Centre for Rural Health Service, Wuhan, China

**Keywords:** China, healthcare utilization, migrants, middle-aged, older adults

## Abstract

**Background:**

An increasing number of middle-aged and older adults are migrating from rural to urban areas for employment, to care for their younger generation and due to old age. As these age groups move into urban areas, their healthcare service utilization are directly related to their health status and basic rights to survival. It also places higher demands on China’s healthcare service provision.

**Objectives:**

This study aims to examine effects of rural-to-urban migration on healthcare utilization among middle-aged and older adults in China.

**Methods:**

Panel data from Waves 3, 4, and 5 of the China Health and Retirement Longitudinal Study were used. We included 318 participants in the exposure group and 7,525 participants in the control group. The study employed propensity score matching (PSM) and Difference in Difference (DID) analysis.

**Results:**

Difference in difference regression results showed that middle-aged and older adults reduced number of hospitalizations when they moved to a city with DID values of −0.092 (*p* < 0.10) for the period 2015–2018 and − 0.135 (*p* < 0.05) for the period 2015–2020. No significant effects were observed regarding the number of outpatient visits (*p* > 0.05).

**Conclusion:**

Middle-aged and older migrants who migrated to cities reduced inpatient healthcare utilization, possibly due to lower hospitalization reimbursement rates, financial burden, and lower social integration. Policies enhancing health insurance reimbursement rates for migrants, integrated community support programs, and strengthening health education to promote health equity may provide remedy.

## Introduction

In recent years, an increasing number of middle-aged and older adults have been migrating from rural to urban areas or from economically underdeveloped to economically developed areas (1). This trend is driven by various factors, including global industrialization and urbanization, better employment opportunities, expectations of higher income, and improved social services (2). Since 2015, the Chinese migrant population has stabilized at around 245 million, while the number of older migrants continues to rise (3). Compared to younger migrants, middle-aged and older adults are more likely to have diagnosed chronic diseases and higher healthcare utilization (4). Despite the potential for improved healthcare access in urban areas, many middle-aged and older migrants continue to face significant barriers. These include geographic distance from health insurance schemes, higher out-of-pocket medical expenses, and limited access to specialized medical services (5). These challenges highlight the urgent need to understand the healthcare utilization patterns of middle-aged and older migrants, particularly in the context of their migration from rural to urban areas.

To better understand these challenges, previous studies have explored various aspects of migrants’ health and healthcare access. Most existing research has focused on health status (6), health awareness (7), inequalities (8), and respective determinants in migrant populations. Corresponding evidence suggests that migrants’ health is shaped by individual characteristics such as age, gender, education, and marital status (9). Additionally, the timing and extent of migration significantly impact migrants’ health outcomes (10). A key structural barrier is China’s household registration system (hukou), which ties health insurance benefits to an individual’s registered residence, resulting in higher out-of-pocket costs and complex reimbursement procedures for rural-to-urban migrants (2). Research from Germany indicated, for instance, that immigrants utilize healthcare services less frequently and face greater inequalities than non-immigrants (11); and a study from Switzerland found that regularizing the status of undocumented migrants increased their utilization of health services (12). However, most previous studies have utilized cross-sectional data or compared migrant populations with residents (8, 13), leaving a significant gap in understanding how healthcare utilization changes before and after urban relocation among middle-aged and older migrants.

This study aims to close this gap by evaluating the effects of rural-to-urban migration on healthcare utilization among middle-aged and older adults using panel data from the China Health and Retirement Longitudinal Study (CHARLS) for the years 2015, 2018, and 2020. Specifically, we hypothesize that middle-aged and older migrants who move from rural to urban areas will experience a decrease in the number of outpatient visits (NOO) and hospitalizations (NOH). The findings will inform strategies to improve the health of middle-aged and older migrants and guide the development of health insurance policies, thereby promoting healthy aging and advancing health equity.

## Methods

### Data sources

CHARLS aims to collect high-quality microdata that are representative of Chinese households and adults aged 45 years and older. Being a nationally representative survey of the older population, it covers 150 district units, 450 village units, and approximately 17,000 adults in 10,000 households at baseline (14). Participants are followed up every 2–3 years. CHARLS collects data on consumption, work, income and assets, health status, health insurance, and information on type of residence and health services utilization. Overall, the CHARLS survey provides a comprehensive depiction of the older population in China. To enhance the representativeness of our findings, we applied longitudinal sampling weights from the CHARLS database, which account for non-response at both household and individual levels. Additionally, we used multiple imputation with chained equations and performed 5 imputations to address item-missing data.

### Sample selection

**Figure 1:**
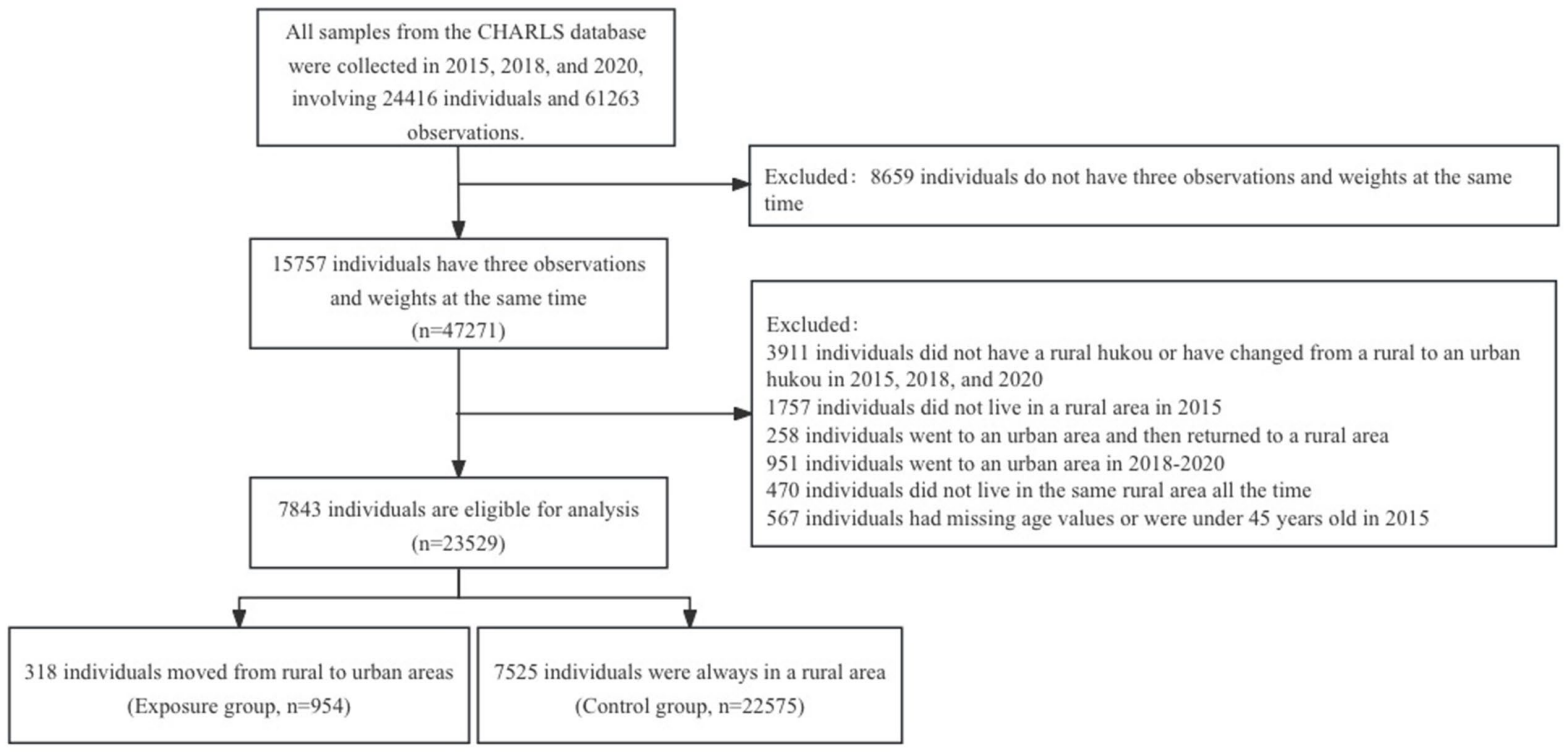
Flowchart of the data selection process (China, 2015 to 2020).

This longitudinal study used data from the third (2015), fourth (2018), and fifth (2020) waves of CHARLS. The data were matched by respondent ID to include participants surveyed in all three waves. Next, we excluded participants who lived in towns, moved from urban to rural areas, or lived in combined townships in 2015, were younger than 45 years old, or had missing data regarding date of birth. Additionally, participants with non-agricultural hukou (household registration), those who did not live in the same rural area all the time, those who returned to rural areas after migrating to cities, and those who migrated to cities after 2015, that is during 2018–2020, were also excluded. This process resulted in 23,529 observations for 7,843 adults, divided into the rural-to-urban group (exposure group, 954 observations for 318 adults) and the group that remained in rural areas (control group, 22,575 observations for 7,525 adults). Figure 1 illustrates the data selection process.

### Variables

Outcomes of this study were NOO in the previous month and NOH in the last year. NOO represents the total number of outpatient visits to various medical institutions over the last month, while NOH represents the total number of inpatient visits over the past year.

Region was set as an independent variable based on two questions from CHARLS’ basic information section: ‘What is your current address?’, and ‘Is it a village or a city/town?’. Participants who lived in towns in 2015 were excluded, ensuring that all participants lived in villages at the present study’s baseline.

For control variables, the Anderson model of healthcare utilization was consulted. This model includes predisposing, enabling, and need factors and is a well-established framework for analyzing healthcare service use with a behavioral measurement model (15, 16). This study began by selecting covariates that may influence rural-to-urban migration and healthcare utilization, guided by the Anderson service model and relevant literature (17, 18). Predisposing factors included age, gender (male = 1, female = 0), education (illiterate = 1, primary = 2, junior high school = 3, senior high school = 4, bachelor’s degree and above = 5), and marital status (divorced, widowed, and never married = 0, married or cohabitation = 1). Enabling factors consisted of economic status (measured by per capita household expenditure, which is logarithmic to reduce the effect of outliers and make the results more stable) and health insurance (no = 1, employee health insurance = 2, urban and rural resident health insurance = 3, others = 4), social activities (participating in social activities = 1, none = 0), household size (number of people living in the household). Need factors included self-assessed health (very good and good = 1, fair, poor, and very poor = 0), chronic disease prevalence (with chronic disease = 1, without = 0), smoking (current or previous smoker = 1, never smoked = 0), and alcohol consumption (consumes alcohol = 1, abstinent = 0), activities of daily living (ADL) included dressing, bathing, eating, getting into or out of bed, using the bathroom, controlling urination and defecation (having no difficulty = 0, having difficulty but still able to perform independently =1, having difficulty and needing help = 2, unable to perform activity = 3, with a total score ranging from 0 to 18), and instrumental activities of daily living (IADL) included doing household chores, cooking, shopping, making phone calls, taking medication, managing money (scales as for ADL, with a total score also ranging from 0 to 18). Table 1 presents variables categorized and described.

**Table 1:** Descriptive statistics results (China, 2015).

Variables	Variable definitions and descriptions	(2)	(3)	*P* (difference exposure vs. control)*
		Exposure group (*n* = 318)	Control group (*n* = 7,525)	
Dependent variables				
NOO	Number of outpatient visits	0.40(1.21)	0.45(1.45)	0.54
NOH	Number of hospitalizations	0.21(0.64)	0.19(0.66)	0.38
Control variables				
Gender	Female = 0	180(56.60)	4,059(53.94)	0.35
	Male = 1	138(43.40)	3,466(46.06)	
Age	Continuous variable	57.31(8.98)	60.15(9.47)	< 0.01
Education	Illiterate = 1	131(41.19)	3,990(53.02)	< 0.01
	Primary = 2	74(23.27)	1,751(23.27)	
	Junior high school = 3	82(25.79)	1,394(18.52)	
	Senior high school = 4	31(9.75)	382(5.08)	
	Bachelor’s degree and above = 5	0(0.00)	8(0.11)	
Marital status	Divorced, widowed, and never married = 0	29(9.12)	863(11.47)	0.20
	Married or cohabitation = 1	289(90.88)	6,662(88.53)	
Health insurance	No = 1	55(17.30)	1,355(18.00)	< 0.01
	Employee health insurance = 2	15(4.72)	96(1.28)	
	Urban and rural resident health insurance = 3	244(76.73)	6,024(80.05)	
	Others = 4	4(1.26)	50(0.66)	
Self-assessed health	Fair, poor, and very poor = 0	248(77.99)	5,848(77.71)	0.91
	Very good and good = 1	70(22.01)	1,677(22.29)	
Chronic diseases prevalence	Without = 0	95(29.87)	2,265(30.10)	0.93
	With chronic disease = 1	223(70.13)	5,260(69.90)	
Social activities	None = 0	140(44.03)	3,381(50.91)	0.02
	Participating in social activities = 1	178(55.97)	3,694(49.09)	
Economic status	Logarithm of household expenditure per capita	9.27(1.00)	8.94(1.27)	< 0.01
Household size	Number of household members	2.37(1.00)	2.59(1.18)	< 0.01
Alcohol consumption	Alcohol abstinent = 0	207(65.09)	4,928(65.49)	0.14
	Consumes alcohol = 1	111(34.91)	2,597(34.51)	
Smoking	Never smoked = 0	202(63.52)	4,471(59.42)	0.89
	Smoker or previous smoker = 1	116(36.48)	3,054(40.58)	
ADL	Performance in activities of daily living (0–18)	0.50(1.44)	0.51(1.49)	0.97
IADL	Performance in instrumental activities of daily living (0–18)	0.92(2.31)	1.19(2.62)	0.07

Means are reported for continuous variables (mean, SD); frequencies for categorical variables (*N*, %). *p-values were derived from the Chi-square test for categorical variables and the *t*-test for continuous variables.

### Statistical analysis

We used the PSM-DID method to assess the effect of migration to cities on the NOO and NOH among middle-aged and older adults in the sample. The PSM method effectively addresses issues of endogeneity and self-selection within samples (19–21) and is widely used to evaluate the impacts of health and other policies (22, 23). The PSM-DID can address selection bias by matching treated and control groups on observed covariates, thereby ensuring baseline comparability. Moreover, it relaxes the assumption of parallel trends, which extends the traditional DID framework and makes it more suitable for real-world applications. Additionally, PSM-DID is particularly robust in non-experimental settings, as it provides transparent treatment effect estimates by attributing outcome differences to the treatment rather than pre-existing confounders.

First, propensity scores were calculated. The propensity score is a balancing score: conditional on the propensity score, the distribution of measured baseline covariates is similar between treated and untreated adults. Common support refers to the overlapping region in the propensity score distribution between the exposure and control groups. A balancing test was conducted to detect an equilibrium of covariates for the exposure and control group and to confirm the reduction of sampling bias through matching. Nearest neighbor matching with caliper was employed for our primary analysis, each treated adult was matched with three untreated adults, as frequently mentioned in the literature (24, 25). After matching, the deviation should be less than or equal to 5 percent, or *p* > 0.10, to confirm proper matching. The adequacy of the matching was evaluated based on the mean reduction in bias for each covariate and the overall mean reduction in bias. Samples outside the common support were excluded.

Secondly, DID model was constructed after PSM matching while controlling for individual and time effects. As expressed in Equation 1, the model is specified as follows:

(1) $$\begin{array}{l} Y_{\mathrm{it}}=\beta_{0}+\beta_{1}\mathrm{Treate}d_{i}\times Time_{t}+\beta_{2}X_{1\mathrm{it}}+\beta_{3}X_{2\mathrm{it}}+\cdots +\\ \beta_{k+1}X_{\mathrm{kit}}+\mu_{i}+\lambda_{t}+\varepsilon_{\mathrm{it}}\; \end{array}$$

Where $Y_{\mathrm{it}}$ represents an individual i’s healthcare utilization in year t. $\mathrm{Treate}d_{i}$ is a dummy variable for grouping. It is defined as exposure group = 1 and control group = 0. In the current study, 1 denotes migrating to a city (exposure group), and 0 denotes always living in a rural area (control group). $Time_{t}$ is a time dummy variable that defines 2015 = 0, 2018 = 1, and 2020 = 1. $\beta_{1}$ is the coefficient of the core explanatory variable in this study, which is the net effect of middle-aged and older adults migrating to the city on the utilization of healthcare services. It reflects the change in the difference between the exposure group (those who migrated to urban areas) and the control group (those who remained in rural areas). $\beta_{2}X_{1\mathrm{it}}+\beta_{3}X_{2\mathrm{it}}+\cdots +\beta_{k+1}X_{\mathrm{kit}}$ represent the effect of time-varying covariates on the outcome $Y_{\mathrm{it}}$ . $\varepsilon_{\mathrm{it}}$ is the error term, and $\beta_{0}$ is a constant term. $\mu_{i}$ is an individual fixed effect, and $\lambda_{t}$ is a time-fixed effect. Statistical analyses were conducted using Stata 16.0. To validate the robustness of the DID results after PSM, we conducted a comparative analysis using the radius matching method and performed placebo tests. Furthermore, we conducted heterogeneity analyses to examine the differential impacts of rural-to-urban migration on healthcare utilization across different demographic groups. Chronic diseases, self-assessed health, and marital status were selected for heterogeneity analyses because chronic diseases influence healthcare needs, self-assessed health is a key predictor of healthcare utilization, and marital status affects social support and access to resources. These variables were chosen to provide a comprehensive understanding of the differences in healthcare utilization among rural-to-urban migrants, while also considering data availability and study scope constraints.

## Results

Table 1 presents descriptive statistics for main study variables at the 2015 baseline, prior to rural to urban migration of the exposure group. Exposure and control group exhibited differences in age, education, social activities, per capita household expenditure, health insurance, IADL, and household size. Specifically, the mean age of the exposure group was approximately 2.8 years younger than that of the control group. The exposure group further had a higher average education level, engaged in more social activities, had higher per capita household expenditure, and reported better ability to perform instrumental activities of daily living. Moreover, more people in the exposure group were enrolled in employee health insurance and other insurance schemes than in the control group. In contrast, the exposure group’s average household size was smaller than that of the control group. Although there were no significant differences in self-assessed health between exposure and control groups initially, further analysis (see Table 2) revealed that by 2020, middle-aged adults in the exposure group reported poorer self-assessed health compared to the control group. In addition, the exposure group showed reduced participation in social activities after a period of migration to urban areas compared to the control group. In 2018, the exposure group exhibited slightly better ADL than the control group, while by 2020, there were no significant differences between the two groups. Similarly, the exposure group performed better in IADL in 2018; by 2020, the gap between the two groups gradually narrowed, with the exposure group showing a slight advantage in IADL.

**Table 2:** Comparison of self-assessed health, social activities, ADL, and IADL between the exposure and control group (China, 2018 and 2020).

Variables	2018		*P* (difference exposure vs. control)*	2020		*P* (difference exposure vs. control)*
	Exposure group	Control group		Exposure group	Control group	
Self-assessed health (45–59 years old)						
Fair, poor, and very poor	132(78.57)	2,146(74.71)	0.26	123(95.70)	1,887(93.87)	0.08
Very good and good	36(21.43)	725(25.29)		28(4.30)	623(6.13)	
Self-assessed health (≥60 years old)						
Fair, poor, and very poor	122(81.33)	3,779(81.20)	0.97	131(78.44)	4,107(81.89)	0.26
Very good and good	28(18.67)	875(18.80)		36(21.56)	908(18.11)	
Social activities						
None	158(49.69)	3,900(51.83)	0.45	190(59.75)	4,138(54.99)	0.09
Participating in social activities	160(50.31)	3,625(48.17)		128(40.25)	3,387(45.01)	
ADL	0.45(1.69)	0.62(1.71)	0.08	0.69(2.34)	0.83(2.19)	0.25
IADL	1.01(2.78)	1.55(3.16)	<0.01	1.14(3.11)	1.47(3.36)	0.08

Means are reported for continuous variables (mean, SD); frequencies for categorical variables (*N*, %). *P-values were derived from the Chi-square test for categorical variables and the *t*-test for continuous variables.

### PSM results

Table 3 displays the results of the balance test using 1:3 nearest neighbor matching with a caliper. We performed caliper nearest-neighbor matching on the sample from the baseline of 2015 (i.e., before migration). As shown in Table 3, the standard error for each control variable was less than 10% after matching, and no significant differences were found at the 5% level between the means of the variables for the two samples. Additionally, the likelihood ratio chi-square test results using PSM were significantly smaller than those of the original outcomes, further indicating that PSM enhanced overall balance after matching. The matched samples were then aligned with 2018 and 2020 data using respondents’ IDs. Consequently, the final sample included 954 observations in the exposure group and 2,661 observations in the control group.

**Table 3:** Results of balance test after matching (China, 2015).

Variables	Matched	Mean		% bias	% reduction	*t*-test	*p* > |*t*|
		Treated	Control		|Bias|	*t*	
Household size	M	2.37	2.37	−0.10	99.70	−0.01	0.99
Gender	M	0.43	0.47	−6.30	−19.40	−0.80	0.43
Age	M	57.31	57.35	−0.50	98.50	−0.06	0.95
Education	M	2.04	2.10	−6.10	78.70	−0.73	0.47
Marital status	M	0.91	0.91	1.00	86.60	0.14	0.89
Self-assessed health	M	0.22	0.21	3.30	−380.60	0.42	0.68
Chronic diseases prevalence	M	0.70	0.69	1.40	−172.40	0.17	0.86
Social activities	M	0.56	0.56	0.00	100.00	−0.00	1.00
Economic status	M	9.27	9.24	2.60	91.10	0.37	0.71
Smoking	M	0.36	0.39	−5.20	39.00	−0.65	0.51
Alcohol consumption	M	0.35	0.37	−4.20	−437.40	−0.52	0.60
Health insurance	M	2.62	2.60	1.90	−5.80	0.23	0.82
ADL	M	0.51	0.51	0.10	40.20	0.02	0.96
IADL	M	0.92	0.94	−0.80	92.90	−0.11	0.91

### Parallel trend test

A fundamental assumption of the DID approach is that the exposure and control groups follow parallel trends in the outcome variable prior to the intervention. This requires at least two pre-treatment periods for a formal parallel trend test. However, as this study only includes one pre-treatment period, we follow the approach used in previous research (26), which considers the absence of a significant difference in the outcome variable between the exposure and control groups, after controlling for other factors, as indicative of parallel trends. As shown in Table 4, after adjusting for potential confounders, there is no statistically significant difference in outpatient visits and hospitalizations between the exposure and control groups before migration, suggesting that the parallel trend assumption holds. Therefore, the DID model is appropriate for this analysis.

**Table 4:** Results of parallel trend test (China, 2015).

Variables	NOO	NOH
Treated	−0.060(0.081)	0.035(0.040)
Control variables	Yes	Yes
*R* ^2^	0.043	0.077
Number of observations	1,205	1,205

***p < 0.01, **p < 0.05, *p < 0.1; values in parentheses represent robust standard errors.

### DID results

First, the DID method was applied to analyze NOO and NOH using the 2015 and 2018 data. The results revealed no significant negative effect on NOO and NOH for middle-aged and older adults migrating to cities (see Table 5). Second, NOO and NOH were analyzed using DID for the 2015 and 2020 data. The DID coefficient for NOH was −0.135, with a *p*-value<0.05, indicating that compared to the control group, middle-aged and older adults in the exposure group had 0.135 fewer hospitalizations after migrating to an urban area. In contrast, the DID coefficient for NOO was −0.032 with a *p*-value > 0.05 for 2015–2020. These findings suggest that migration to cities did not significantly affect NOO for middle-aged and older adults.

**Table 5:** Difference-in-differences estimate results (China, 2015–2018 and 2015–2020).

Variables	NOO	NOH	NOO	NOH
	2015–2018	2015–2018	2015–2020	2015–2020
DID	−0.015(0.109)	−0.092(0.053)*	−0.032(0.123)	−0.135**(0.057)
Control variables	Yes	Yes	Yes	Yes
Time fixed effects	Yes	Yes	Yes	Yes
Individual fixed effects	Yes	Yes	Yes	Yes
*R* ^2^	0.579	0.689	0.561	0.667
Number of observations	2,410	2,410	2,410	2,410

***p < 0.01, **p < 0.05, *p < 0.1; values in parentheses represent robust standard errors.

### Sensitivity analyses

#### Replacement of the matching method

To ensure the robustness of the PSM-DID results, an alternative matching method, specifically radius matching, was employed. The DID regressions were re-estimated using this different matching method, as presented in Table 6. The results remained consistent with the initial findings, suggesting that the empirical analyses demonstrate strong reliability.

**Table 6:** Difference-in-differences estimation results after changing the matching method (China, 2015 -2020).

Variables	NOO	NOH
	2015–2020	2015–2020
DID	−0.020(0.098)	−0.130***(0.044)
Control variables	Yes	Yes
Time fixed effects	Yes	Yes
Individual fixed effects	Yes	Yes
*R* ^2^	0.561	0.596
Number of observations	15,322	15,322

***p < 0.01, **p < 0.05, *p < 0.1; values in parentheses represent robust standard errors.

#### Placebo testing

**Figure 2:**
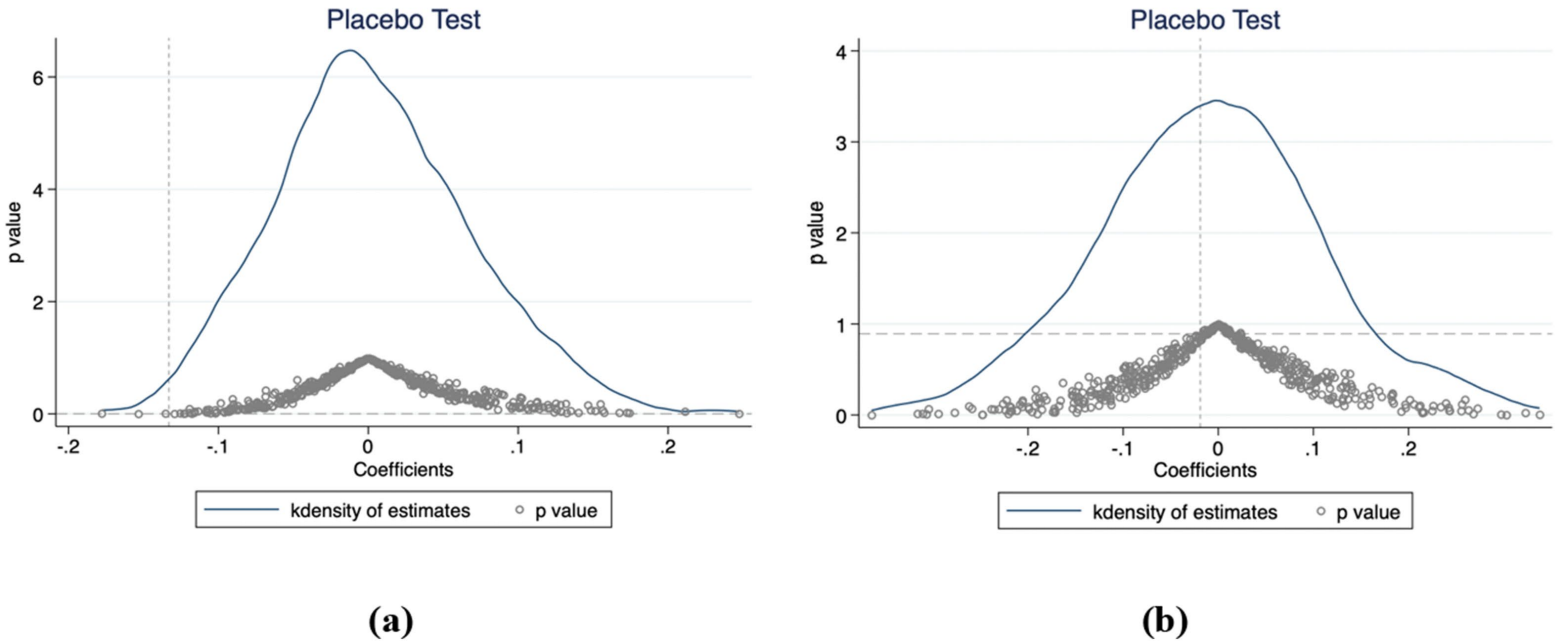
Placebo test results for the coefficient on the number of hospitalizations **(a)** and outpatient visits **(b)** (China, 2015–2020).

To control for the potential bias arising from omitted variables in the above results, referring to a previous study (27), a placebo test was performed. The placebo test is a regression in which any of the dummy exposure groups in the sample are randomly selected and the process is repeated 500 times. The results of the placebo test for 2015–2020 are shown in Figure 2. Figure 2a displays the estimated coefficients for the number of hospitalizations, which are centered around zero and follow a normal distribution, indicating a robust model. The dotted lines represent the true estimated coefficients, which are far from these virtual estimates, indicating that the result is not sensitive to unobserved factors or the effects of random interventions. Figure 2b displays the estimated coefficients for the number of outpatient visits. The dotted line remains insignificant within the normal value range, demonstrating the robustness of the study’s results.

#### Winsorization

To enhance the robustness of the results, we used the Winsorization process. The sample values for the lowest and highest 1% of each of the number of hospitalizations and outpatient visits were replaced with 1st percentile and 99th percentile values. Through this process, we aim to minimize the impact of outliers, resulting in more robust results. This method helps to ensure that estimates of NOO and NOH more accurately reflect respondents’ healthcare attendance and avoid bias in the results due to extreme observations. As shown in Table 7, the results are consistent with the previous findings.

**Table 7:** The results of winsorization (China, 2015–2020).

Variables	NOO	NOH
	2015–2020	2015–2020
DID	−0.045(0.087)	−0.098**(0.046)
Control variables	Yes	Yes
Time fixed effects	Yes	Yes
Individual fixed effects	Yes	Yes
*R* ^2^	0.587	0.618
Number of observations	2,390	2,390

***p < 0.01, **p < 0.05, *p < 0.1; values in parentheses represent robust standard errors.

#### Excluding the impact of the COVID-19 pandemic

The global spread of the COVID-19 pandemic in 2020 impacted the utilization of medical services. To account for this, we excluded responses to the survey question, “During the pandemic, were there any medical services or treatments you needed, such as hospitalization for major surgeries or outpatient care, that were delayed or canceled?” Specifically, we removed samples indicating the need for “major surgeries requiring hospitalization” and “outpatient services” affected by delays or cancelations. After excluding these cases, the results remained consistent with the above findings (Table 8).

**Table 8:** Results after excluding samples affected by the COVID-19 pandemic (China, 2015–2020).

Variables	NOO	NOH
DID	−0.044(0.129)	−0.149**(0.595)
Control variables	Yes	Yes
Time fixed effects	Yes	Yes
Individual fixed effects	Yes	Yes
*R* ^2^	0.589	0.626
Number of observations	2,192	2,350

***p < 0.01, **p < 0.05, *p < 0.1; values in parentheses represent robust standard errors.

### Heterogeneity analyses

Heterogeneity analyses indicated that healthcare utilization varied among different subgroups of middle-aged and older migrants. These analyses revealed how rural-to-urban migration affected healthcare service utilization across various self-assessed health conditions, age, and chronic disease groups, providing valuable insights for policymakers to formulate more targeted recommendations. Table 9 showed a significant reduction in NOH for migrants with chronic diseases who moved to cities. Additionally, the same table highlighted a significant reduction in NOH for migrants who self-assessed their health as fair, poor, or very poor following their migration to urban areas. Furthermore, Table 9 indicated that 60–74-year-olds who moved to cities had fewer hospitalizations.

**Table 9:** Results of the test for heterogeneity in the number of hospitalizations (China, 2015–2020).

Variables	Chronic diseases		Self-assessed health		Age		
	With chronic disease	Without	Very good and good	Fair, poor and very poor	45–59	60–74	75-
DID	−0.135* (0.075)	0.081 (0.074)	−0.034 (0.058)	−0.180** (0.077)	−0.047 (0.062)	−0.225** (0.114)	0.176 (0.319)
Control variables	Yes	Yes	Yes	Yes	Yes	Yes	Yes
Time fixed effects	Yes	Yes	Yes	Yes	Yes	Yes	Yes
Individual fixed effects	Yes	Yes	Yes	Yes	Yes	Yes	Yes
*R* ^2^	0.682	0.637	0.519	0.668	0.604	0.689	0.679
Number of observations	1,632	328	254	1,696	1,090	710	108

***p < 0.01, **p < 0.05, *p < 0.1; values in parentheses represent robust standard errors.

## Discussion

At present, few studies have used longitudinal data to examine healthcare utilization among middle-aged and older rural-to-urban migrants in China. While previous research has primarily relied on cross-sectional data or compared migrants with residents (8, 13), this study provides novel insights by tracking changes in healthcare utilization before and after migration. Consistent with previous studies (28, 29), our research demonstrated that rural middle-aged and older migrants who moved to urban areas experienced a reduction in hospitalization services utilization. However, our study found that this effect becomes more pronounced with longer durations of urban residence and further extended this understanding by revealing nuanced heterogeneity effects based on individual characteristics. Specifically, we found that migration to urban areas has a more pronounced impact on healthcare utilization among adults with chronic diseases, those with fair or poor self-assessed health, and those aged 60–74. However, contrary to the findings of the previous study (30), the change in residence did not significantly affect the number of outpatient visits, which remains stable. These findings highlight the importance of considering demographic and health status variations when designing policies for migrant populations, a point that has been largely overlooked in prior research.

First, the reduction of hospitalizations may be attributed to healthcare policies related to reimbursement for medical treatment outside one’s registered area (8, 29, 31). Despite ongoing improvements in China’s reimbursement policies, the rates for medical treatment outside one’s enrolment area remain lower than those for local treatment (32). For example, in Qingyuan City, Guangdong Province, the reimbursement rates for hospitalization are 90% for level 1 hospitals, 75% for level 2, and 65% for level 3 if the treatment is received within the enrolment area. In contrast, when treatment is sought outside the enrolment area, the reimbursement rates drop to 70% for level 1, 60% for level 2, and 50% for level 3 (33). Consequently, the reimbursement rates for equivalent medical services are reduced by 15–20 percentage points when treatment is provided elsewhere. Furthermore, before the implementation of direct remote settlement of medical expenses, China’s reimbursement process was complex, requiring upfront payment and subsequent reimbursement at the enrolment location. Similarly, a study conducted in Kenya found that despite national treaties advocating for equal healthcare rights, immigrants often face higher healthcare costs than local residents (34). In the Greater Mekong Subregion, migrants encounter elevated health risks but are inadequately covered, leading to high out-of-pocket payments for health services (35). When designing a high-quality universal health coverage system, it is essential to enable middle-aged and older migrants to enroll in insurance at their place of migration, expand the level and scope of reimbursement for medical treatment in other locations, and reduce the disparity between treatments received within and outside the insured area.

Second, according to Anderson’s model, enabling factors such as financial resources and social capital influence healthcare utilization. Relevant studies indicate that most rural-to-urban migrants forego hospitalization because of poor affordability (36, 37). This is particularly true for the middle-older age group (60–74 years), who may be between retirement and intergenerational care. Compared to their rural counterparts, this group is more susceptible to chronic diseases (38), they tend to prioritize financial stability over personal health to avoid burdening their children, often choosing to overlook their illnesses (13, 39). Although their presence in urban areas increases demand for healthcare services, these adults often underutilize healthcare services due to financial difficulties and restrictive insurance policies (40). Furthermore, lower levels of social integration among middle-aged and older migrants may lead to negative attitudes toward healthcare and inadequate access to preventive and primary public health services (41, 42). To alleviate the financial burden associated with hospitalization, policymakers should consider modest improvements in healthcare treatment for vulnerable migrants. Addressing these challenges also requires fostering a supportive environment at both community and household levels to facilitate the integration of middle-aged and older migrants into urban communities (43, 44).

Third, the effect of moving to an urban area on hospitalization among middle-aged and older adults may take time to become significant. This delay could be attributed to the fact that some adults in these groups have recently migrated and may still be adapting to the new living environment and healthcare system. As a result, they may continue to rely on their established habits for seeking medical care. Steventon similarly observed that hospital admission rates among migrants remained low for several years following their arrival in the UK (45). However, contrary to the findings of the previous study (30), migration to cities did not significantly affect the NOO among these age groups. This may be due to the low reimbursement rates and limited caps on outpatient service reimbursements under many Chinese insurance schemes, which lead to high out-of-pocket costs for middle-aged and older adults. Policymakers should consider enhancing the medical insurance system for treatments received in other locations, integrating community support programs, and promoting health literacy to improve health outcomes for migrant populations (46, 47).

Several limitations should be acknowledged. Firstly, the implementation of PSM-DID relies on the assumption that all relevant covariates are included in the model. Any unobserved covariates can introduce bias by causing different trends between the treatment and control groups. Future research should consider using alternative methods to mitigate the risk of bias. Additionally, the limitations of the CHARLS database restrict our ability to perform in-depth analyses of migration destinations. Adults are nested within provinces, and the development of healthcare services varies significantly across different regions of China, complicating the analysis of healthcare utilization patterns. Future research should employ alternative methods and utilize more detailed datasets that incorporate a broader range of health assessments and regional characteristics to enhance our understanding of healthcare utilization among rural-to-urban migrants.

## Conclusion

This study demonstrates that migration from rural to urban areas significantly reduces hospital admissions among middle-aged and older migrants. However, utilization of outpatient services remains unchanged. These findings highlight the need for policymakers to address the specific healthcare challenges faced by this demographic group. In China, where rapid urbanization and economic transformation have led to substantial internal migration, policymakers should enhance the medical insurance system for treatment outside the registered area, develop community support programs, and strengthen health education to improve the well-being of middle-aged and older migrants while ensuring equitable access to care.

## Data Availability

The original contributions presented in the study are publicly available. This data can be found at: https://charls.pku.edu.cn/en/.

## References

[1] Xing Y Zhang L Zhang Y He R. Relationship between social interaction and health of the floating elderly population in China: an analysis based on interaction type, mode and frequency. BMC Geriatr. (2023) 23:662. doi: 10.1186/s12877-023-04386-z, PMID: 37845627 PMC10580520

[2] Gong P Liang S Carlton EJ Jiang Q Wu J Wang L. Urbanisation and health in China. Lancet. (2012) 379:843–52. doi: 10.1016/S0140-6736(11)61878-3, PMID: 22386037 PMC3733467

[3] Gao L Penning MJ Wu Z Sterrett SJ Li S. Internal migration and the health of middle-aged and older persons in China: the healthy migrant effect reconsidered. Res Aging. (2021) 43:345–57. doi: 10.1177/0164027520958760, PMID: 32964791

[4] Zhong BL Liu TB Chan SSM Jin D Hu CY Dai J. Common mental health problems in rural-to-urban migrant workers in Shenzhen, China: prevalence and risk factors. Epidemiol Psychiatr Sci. (2018) 27:256–65. doi: 10.1017/S2045796016001141, PMID: 28067189 PMC6998856

[5] Xie Y Guo Q Meng Y. The health service use of aged rural-to-urban migrant workers in different types of cities in China. BMC Health Serv Res. (2021) 21:606. doi: 10.1186/s12913-021-06638-3, PMID: 34182984 PMC8237433

[6] Fu Y Lin W Yang Y Du R Gao D. Analysis of diverse factors influencing the health status as well as medical and health service utilization in the floating elderly of China. BMC Health Serv Res. (2021) 21:438. doi: 10.1186/s12913-021-06410-7, PMID: 33964906 PMC8106125

[7] Hai Y Wu WL Yu LW Wu L. Health literacy and health outcomes in China's floating population: mediating effects of health service. BMC Public Health. (2021) 21:691. doi: 10.1186/s12889-021-10662-7, PMID: 33832480 PMC8030651

[8] Xi S Song Y Li X Li M Lu Z Yang Y. Local-migrant gaps in healthcare utilization between older migrants and local residents in China. J Am Geriatr Soc. (2020) 68:1560–7. doi: 10.1111/jgs.16421, PMID: 32222105

[9] Wang Q. Health of the elderly migration population in China: benefit from individual and local socioeconomic status? Int J Environ Res Public Health. (2017) 14:370. doi: 10.3390/ijerph14040370, PMID: 28368314 PMC5409571

[10] Niu L Liu Y Wang X. Using nomogram to predict the hospitalization forgone among internal migrants in China: a nationally representative cross-sectional secondary data analysis. Risk Manag Healthc Policy. (2021) 14:3945–54. doi: 10.2147/RMHP.S301234, PMID: 34584472 PMC8464368

[11] Klein J von dem Knesebeck O. Inequalities in health care utilization among migrants and non-migrants in Germany: a systematic review. Int J Equity Health. (2018) 17:160. doi: 10.1186/s12939-018-0876-z 30382861 PMC6211605

[12] Fakhoury J Burton-Jeangros C Consoli L Duvoisin A Jackson Y. Association between residence status regularization and access to healthcare for undocumented migrants in Switzerland: a panel study. Front Public Health. (2022) 10:832090. doi: 10.3389/fpubh.2022.832090, PMID: 35664122 PMC9160788

[13] Wang Y Jing Z Ding L Tang X Feng Y Li J. Socioeconomic inequity in inpatient service utilization based on need among internal migrants: evidence from 2014 national cross-sectional survey in China. BMC Health Serv Res. (2020) 20:984. doi: 10.1186/s12913-020-05843-w, PMID: 33109188 PMC7590715

[14] Zhao Y Hu Y Smith JP Strauss J Yang G. Cohort profile: the China health and retirement longitudinal study (CHARLS). Int J Epidemiol. (2014) 43:61–8. doi: 10.1093/ije/dys203 23243115 PMC3937970

[15] Andersen RM. Revisiting the behavioral model and access to medical care: does it matter? J Health Soc Behav. (1995) 36:1–10. doi: 10.2307/2137284, PMID: 7738325

[16] Peng B Ling L. Health service behaviors of migrants: a conceptual framework. Front Public Health. (2023) 11:1043135. doi: 10.3389/fpubh.2023.1043135, PMID: 37124818 PMC10140430

[17] Zhou M Zhu W Sun X Huang L. Internet addiction and child physical and mental health: evidence from panel dataset in China. J Affect Disord. (2022) 309:52–62. doi: 10.1016/j.jad.2022.04.115, PMID: 35469911

[18] Xie L Yao Y-D Tang L-l Zhang S Yang H-L Zhang S-Q. Effect of working after retirement on the mental health of older people: evidence from China. Front Psych. (2021) 12:12. doi: 10.3389/fpsyt.2021.731378 PMC8505967 34650455

[19] Nolan A. Evaluating the impact of eligibility for free care on the use of general practitioner (GP) services: a difference-in-difference matching approach. Soc Sci Med. (2008) 67:1164–72. doi: 10.1016/j.socscimed.2008.06.021, PMID: 18640757

[20] Baser O. Too much ado about propensity score models? Comparing methods of propensity score matching. Value Health. (2006) 9:377–85. doi: 10.1111/j.1524-4733.2006.00130.x, PMID: 17076868

[21] Austin PC. An introduction to propensity score methods for reducing the effects of confounding in observational studies. Multivariate Behav Res. (2011) 46:399–424. doi: 10.1080/00273171.2011.568786, PMID: 21818162 PMC3144483

[22] Su D Chen YC Gao HX Li HM Chang JJ Jiang D. Effect of integrated urban and rural residents medical insurance on the utilisation of medical services by residents in China: a propensity score matching with difference-in-differences regression approach. BMJ Open. (2019) 9:e026408. doi: 10.1136/bmjopen-2018-026408 PMC6377539 30782944

[23] Yang S Guo D Bi S Chen Y. The effect of long-term care insurance on healthcare utilization of middle-aged and older adults: evidence from China health and retirement longitudinal study. Int J Equity Health. (2023) 22:228. doi: 10.1186/s12939-023-02042-x, PMID: 37904167 PMC10617164

[24] Ma X Feng W Shi C Wang Y Gao Q Cai W. Association between the location of social medical insurance and social integration among China’s elderly rural migrants: a nationwide cross-sectional study. BMC Public Health. (2023) 23:2108. doi: 10.1186/s12889-023-16956-2, PMID: 37884916 PMC10604806

[25] Antu JF Parvin K Sujan HM Mamun MA Naved RT. Effect of rural-urban migration on age at marriage among adolescent girls in Bangladesh. Front Public Health. (2022) 10:840145. doi: 10.3389/fpubh.2022.840145, PMID: 35874980 PMC9298772

[26] Li P Lu Y Wang J. Does flattening government improve economic performance? Evidence from China. J Dev Econ. (2016) 123:18–37. doi: 10.1016/j.jdeveco.2016.07.002, PMID: 40128009

[27] He Y Ouyang W Li Z Wei B. Impact of the industrialization of older adult care services on older Individuals' physical and mental health: evidence from China's quasi-natural experiment. J Multidiscip Healthc. (2023) 16:3017–33. doi: 10.2147/JMDH.S426710, PMID: 37869611 PMC10588744

[28] Shaaban AN Morais S Peleteiro B. Healthcare services utilization among migrants in Portugal: results from the National Health Survey 2014. J Immigr Minor Health. (2019) 21:219–29. doi: 10.1007/s10903-018-0744-3, PMID: 29644552

[29] Zhang X Yu B He T Wang P. Status and determinants of health services utilization among elderly migrants in China. Glob Health Res Policy. (2018) 3:8. doi: 10.1186/s41256-018-0064-0, PMID: 29568805 PMC5861660

[30] Li D Yang J Liu H Ma Y Jiang J. Comparing income-related inequality on health service utilisation between older rural-to-urban migrant workers and older rural residents in China: a cross-sectional study. BMJ Open. (2023) 13:e060581. doi: 10.1136/bmjopen-2021-060581, PMID: 36731937 PMC9896347

[31] Han J Meng Y. Institutional differences and geographical disparity: the impact of medical insurance on the equity of health services utilization by the floating elderly population – evidence from China. Int J Equity Health. (2019) 18:91. doi: 10.1186/s12939-019-0998-y 31200716 PMC6570924

[32] Chen S Chen Y Feng Z Chen X Wang Z Zhu J. Barriers of effective health insurance coverage for rural-to-urban migrant workers in China: a systematic review and policy gap analysis. BMC Public Health. (2020) 20:408. doi: 10.1186/s12889-020-8448-8, PMID: 32228665 PMC7106835

[33] Qingyuan Municipal People's Government. How is medical insurance reimbursement for off-site treatment handled for urban and rural residents? (2024). Available online at: https://www.gdqy.gov.cn/gdqy/newxxgk/rdhy/content/post_1858168.html (Accessed September 10, 2024)

[34] Arnold C Theede J Gagnon A. A qualitative exploration of access to urban migrant healthcare in Nairobi, Kenya. Soc Sci Med. (2014) 110:1–9. doi: 10.1016/j.socscimed.2014.03.019, PMID: 24698760

[35] McMichael C Healy J. Health equity and migrants in the greater Mekong subregion. Glob Health Action. (2017) 10:1271594. doi: 10.1080/16549716.2017.1271594, PMID: 28452652 PMC5328359

[36] Zhang X Wu Q Shao Y Fu W Liu G Coyte PC. Socioeconomic inequities in health care utilization in China. Asia Pacific J Public Health. (2015) 27:429–38. doi: 10.1177/1010539514565446, PMID: 25563350

[37] Song X Zou G Chen W Han S Zou X Ling L. Health service utilisation of rural-to-urban migrants in Guangzhou, China: does employment status matter? Trop Med Int Health. (2017) 22:82–91. doi: 10.1111/tmi.12801, PMID: 27775826

[38] Li D Zhou Z Shen C Zhang J Yang W Nawaz R. Health disparity between the older rural-to-urban migrant workers and their rural counterparts in China. Int J Environ Res Public Health. (2020) 17:17. doi: 10.3390/ijerph17030955, PMID: 32033086 PMC7038012

[39] Zhong BL Liu TB Huang JX Fung HH Chan SS Conwell Y. Acculturative stress of Chinese rural-to-urban migrant workers: a qualitative study. PLoS One. (2016) 11:e0157530. doi: 10.1371/journal.pone.0157530, PMID: 27300005 PMC4907425

[40] Li L Yang J Zhai S Li D. Determinants of differences in health service utilization between older rural-to-urban migrant workers and older rural residents: evidence from a decomposition approach. Int J Environ Res Public Health. (2022) 19:6245. doi: 10.3390/ijerph19106245, PMID: 35627780 PMC9141272

[41] Li J Rose N. Urban social exclusion and mental health of China's rural-urban migrants – a review and call for research. Health Place. (2017) 48:20–30. doi: 10.1016/j.healthplace.2017.08.009, PMID: 28892746

[42] Tang S Long C Wang R Liu Q Feng D Feng Z. Improving the utilization of essential public health services by Chinese elderly migrants: strategies and policy implication. J Glob Health. (2020) 10:010807. doi: 10.7189/jogh.10.010807, PMID: 32257170 PMC7125420

[43] Eckman MH Wise R Leonard AC Dixon E Burrows C Khan F. Impact of health literacy on outcomes and effectiveness of an educational intervention in patients with chronic diseases. Patient Educ Couns. (2012) 87:143–51. doi: 10.1016/j.pec.2011.07.020, PMID: 21925823

[44] Tian Y Luo T Chen Y. The promotional effect of health education on the medical service utilization of migrants: evidence from China. Front Public Health. (2021) 9:818930. doi: 10.3389/fpubh.2021.818930, PMID: 35155362 PMC8831805

[45] Steventon A Bardsley M. Use of secondary care in England by international immigrants. J Health Serv Res Policy. (2011) 16:90–4. doi: 10.1258/jhsrp.2010.010097, PMID: 21389062

[46] Kim M Gu H. Relationships between health education, health behaviors, and health status among migrants in China: a cross-sectional study based on the China migrant dynamic survey. Healthcare. (2023) 11:11. doi: 10.3390/healthcare11121768, PMID: 37372886 PMC10298536

[47] Li X Yang H Wang H Liu X. Effect of health education on healthcare-seeking behavior of migrant workers in China. Int J Environ Res Public Health. (2020) 17:2344. doi: 10.3390/ijerph17072344, PMID: 32235675 PMC7177837

